# Can radiotherapy be omitted in T1-2N1 breast cancer patients after mastectomy without neoadjuvant therapy?

**DOI:** 10.3389/fonc.2025.1726994

**Published:** 2026-01-23

**Authors:** Jing Hou, Shuangqiang Qian, Chenyi Liao, Chengwen Wu, Xi Zhang, Yanchun Gao

**Affiliations:** Department of Thyroid and Breast Surgery, School of Clinical Medicine, Affiliated Hospital of North Sichuan Medical College/North Sichuan Medical College, Nanchong, Sichuan, China

**Keywords:** breast cancer, neoadjuvant therapy, postmastectomy, radiotherapy, T1-2N1

## Abstract

**Objective:**

To evaluate the necessity of postmastectomy radiotherapy (PMRT) in patients with T1-2N1M0 breast cancer who did not receive neoadjuvant therapy, by assessing its impact on locoregional recurrence (LRR) and overall survival (OS) in the context of contemporary systemic therapies. This meta-analysis aims to provide updated evidence on whether PMRT can be omitted in this specific population.

**Methods:**

Statistical analysis was conducted using Review Manager version 5.4 software, as recommended by the Cochrane Collaboration. HR for LRR and OS were pooled between the PMRT and no-PMRT groups. A fixed-effects model was primarily used, with a random-effects model applied if heterogeneity (I² > 50%) was detected. Bias risk in the included studies was assessed using the Newcastle-Ottawa Scale, and publication bias was evaluated through funnel plot analysis.

**Results:**

In patients with T1-2N1M0 breast cancer, PMRT significantly reduced the risk of LRR (pooled HR = 0.35, 95% CI: 0.23–0.53; p<0.001) and improved OS (pooled HR = 0.65, 95% CI: 0.61–0.69; p<0.001). Subgroup analyses showed consistent benefit for LRR reduction at 5 years (HR = 0.45, 95% CI: 0.35–0.56) and 10 years (HR = 0.33, 95% CI: 0.19–0.57; interaction p=0.33). For OS, a significant 5-year survival improvement was observed (HR = 0.63, 95% CI: 0.59–0.67; p<0.001), but the 10-year benefit was non-significant (HR = 0.80, 95% CI: 0.60–1.07; p=0.14).

**Conclusions:**

This meta-analysis supports the use of postmastectomy radiotherapy in T1-2N1M0 breast cancer patients, demonstrating its significant reduction in LRR and improvement in OS. Future research should integrate molecular subtypes and dynamic risk models to optimize treatment decisions within contemporary systemic therapy frameworks, and prospective studies are needed to assess the long-term safety of PMRT omission in certain subgroups.

**Systematic Review Registration:**

https://www.crd.york.ac.uk/PROSPERO/view/CRD420261287168, identifier CRD420261287168.

## 1 Introduction

Breast cancer is the most common cancer worldwide, with over 2.3 million cases diagnosed in 2022, and its incidence continues to rise, representing a major cause of cancer-related mortality in women globally (1). Treatment of breast cancer relies on precise risk stratification and personalized management, often combining surgery, systemic therapy and, in selected cases, radiotherapy. Postmastectomy radiotherapy (PMRT) has long been an integral component of adjuvant therapy following mastectomy, with the primary objective of eradicating microscopic locoregional disease to reduce recurrence and improve survival outcomes. In patients with high-risk features such as ≥4 positive axillary lymph nodes or primary tumor stage T3-4, the benefits of PMRT are well established, with randomized and meta-analytic evidence demonstrating substantial reductions in locoregional recurrence (LRR) and breast cancer-specific mortality (2).

But for patients with early-stage breast cancer and limited lymph node involvement, especially those with T1-2 primary tumors and 1-3 positive axillary lymph nodes (T1-2N1M0), the use of PMRT has been debated for the past two decades (3–6). This debate comes from the need to balance the potential absolute benefit of radiotherapy against its risks. These risks include cardiac toxicity, pulmonary sequelae, and impaired quality of life.

The Early Breast Cancer Trialists’ Collaborative Group (EBCTCG) published a landmark meta-analysis in 2014. This study showed that for patients with 1-3 positive lymph nodes, PMRT significantly reduced the 10-year LRR from 21.0% to 4.3% (P<0.01). It also improved the 20-year breast cancer-specific survival from 49.4% to 41.5% (P = 0.01) (2). These results formed the basis for international guidelines and clinical practice. They support the use of PMRT for T1-2N1 patients (7–11).

But modern observational studies report lower LRR than the historical cohorts in the EBCTCG analysis. This is true even for T1-2N1 patients who received PMRT (12, 13). This difference shows that in the current clinical setting, the absolute benefit of PMRT may be smaller. A key driver of this change is the widespread use of modern systemic therapy. This includes more effective chemotherapy regimens like taxanes, targeted therapy like trastuzumab for HER2-positive disease, and extended or intensified endocrine therapy (14, 15). These new chemotherapy drugs, targeted drugs, and endocrine drugs have significantly reduced the risk of distant metastasis and LRR.

This raises a question: for all patients in this group, do the benefits of PMRT still outweigh its risks? This situation requires us to re-evaluate the existing evidence in the context of contemporary systemic therapy strategies.

So, we conducted a meta-analysis of studies published between 2015 and 2025. This study aims to answer two key questions. First, in the era of modern systemic therapy, how strong is the association between PMRT and the risks of LRR and overall survival (OS)? Second, does the existing evidence support omitting PMRT in specific, lower-risk subgroups of T1-2N1M0 patients? By integrating the latest data, this work aims to provide updated, evidence-based insights. These insights can guide shared decision-making between clinicians and patients.

## 2 Materials and methods

### 2.1 Search strategy

This study followed the Preferred Reporting Items for Systematic Reviews and Meta-Analyses (PRISMA) guidelines. Searches were conducted across three major databases: MEDLINE via PubMed, Embase via Ovid, and the Cochrane Library, covering the period from January 1, 2015, to January 31, 2025 (search cutoff date: February 28, 2025). A combination of Medical Subject Headings (MeSH) terms was employed (1): “breast neoplasms” AND “radiotherapy” (2); “mastectomy” AND (“neoadjuvant therapy” OR “adjuvant therapy”). Literature screening followed a dual independent assessment model, where two researchers identified and removed duplicates. For studies with duplicate reports from multicenter cohorts, the PRISMA principles for handling duplicate data were applied, prioritizing studies with a baseline sample size of ≥500 patients and a median follow-up time of ≥60 months. Manual screening of reference lists from included studies was performed to identify additional eligible studies, with conflicts resolved by a third researcher. Only peer-reviewed, full-text English-language articles were retained for final analysis.

### 2.2 Inclusion and exclusion criteria

#### 2.2.1 Inclusion criteria

Inclusion Criteria

Studies included in this meta-analysis had to meet all the following criteria:

- Tumor characteristics: Microscopic tumor size ≤5 cm with 1–3 positive axillary lymph nodes (pathological stage T1-2N1);
- No prior neoadjuvant systemic therapy or radiotherapy;
- Inclusion of retrospective or prospective data;
- Published in English.

#### 2.2.2 Exclusion criteria

The primary exclusion criteria were as follows:

- Inability to extract hazard ratios HR for LRR or OS with 95% CI;
- Studies that included patients who had received neoadjuvant therapy;
- Duplicate publications or studies with overlapping data.

### 2.3 Data abstraction

Based on the inclusion criteria, the following parameters were extracted: first author’s name, publication year, country, study type, enrollment period, proportion of patients receiving primary adjuvant therapy, total number of patients, and median follow-up time. Data extraction was performed independently by two investigators using a pre-designed form, with discrepancies resolved by a third senior investigator.

Hazard ratios HR and 95% CI for LRR and OS were directly extracted from the published articles. If multiple adjusted models were reported, the model adjusted for the greatest number of covariates was prioritized. All extracted data were verified by two investigators.

During screening, if two or more studies were identified as potentially deriving from the same database or having overlapping enrollment periods, a joint assessment was conducted. If substantial overlap was confirmed, only the study that best met the predefined inclusion criteria was included.

## 3 Statistical analysis

This meta-analysis was performed using RevMan 5.4. Pooled HR for LRR and OS were calculated primarily using a fixed-effect model, with a random-effects model applied if heterogeneity (I²) exceeded 50%.

Subgroup and sensitivity analyses were conducted to explore heterogeneity and assess robustness. The risk of bias in included retrospective studies was assessed using the Newcastle-Ottawa Scale (NOS). Publication bias was evaluated using funnel plots.

Subgroup Analysis: Subgroup analyses were conducted to explore potential sources of heterogeneity.

Sensitivity Analysis: A sensitivity analysis was performed by sequentially excluding individual studies to assess the robustness of the results.

Risk of Bias Assessment: The NOS was used to evaluate the risk of bias in retrospective studies.

Publication Bias: Publication bias was assessed using funnel plots, with asymmetry considered significant at a level of P < 0.10.

### 3.1 Assessment of the certainty of evidence

To evaluate the quality of evidence, we employed the GRADE (Grading of Recommendations, Assessment, Development, and Evaluation) approach. The evidence for the primary outcomes, LRR and OS, was evaluated based on the following domains: Study design, Risk of bias, Inconsistency, Indirectness, Imprecision, and Other considerations (**Table 1**).

**Table 1:** Quality of evidence.

Certainty assessment							No of patients		Effect	Certainty	Importance
No of studies	Study design	Risk of bias	Inconsistency	Indirectness	Imprecision	Other considerations	Intervention	Control	HR (95% CI)		
LRR											
7	Retrospective Cohort Study	seriousa	not serious	not serious	not serious	publication bias suspectedb	2577	5205	0.35 (0.23,0.53)	◯ ⨁ ⨁ ⨁ Moderate	CRITICAL
OS											
5	Retrospective Cohort Study	seriousa	not serious	not serious	not serious	publication bias suspectedb	2398	4029	0.65(0.61,0.69)	◯ ⨁ ⨁ ⨁ Moderate	CRITICAL

LRR, Local Recurrence Rate; OS, Overall Survival; HR, Hazard Ratio.

a Downgrading one level for the randomization method and for deviation from intended trial interventions being unclear.

b Downgrading one level for potential publication bias.

All included studies were retrospective cohort studies, which have a higher risk of bias compared to randomized controlled trials. Therefore, the initial rating for all outcomes was categorized as “Low.” During the evidence evaluation process, we carefully addressed potential risks of bias (e.g., confounding and selection bias) and heterogeneity across treatment regimens. Ultimately, the quality of evidence for both LRR and OS was upgraded to “Moderate.”

## 4 Results

### 4.1 Study selection and characteristics

**Figure 1:**
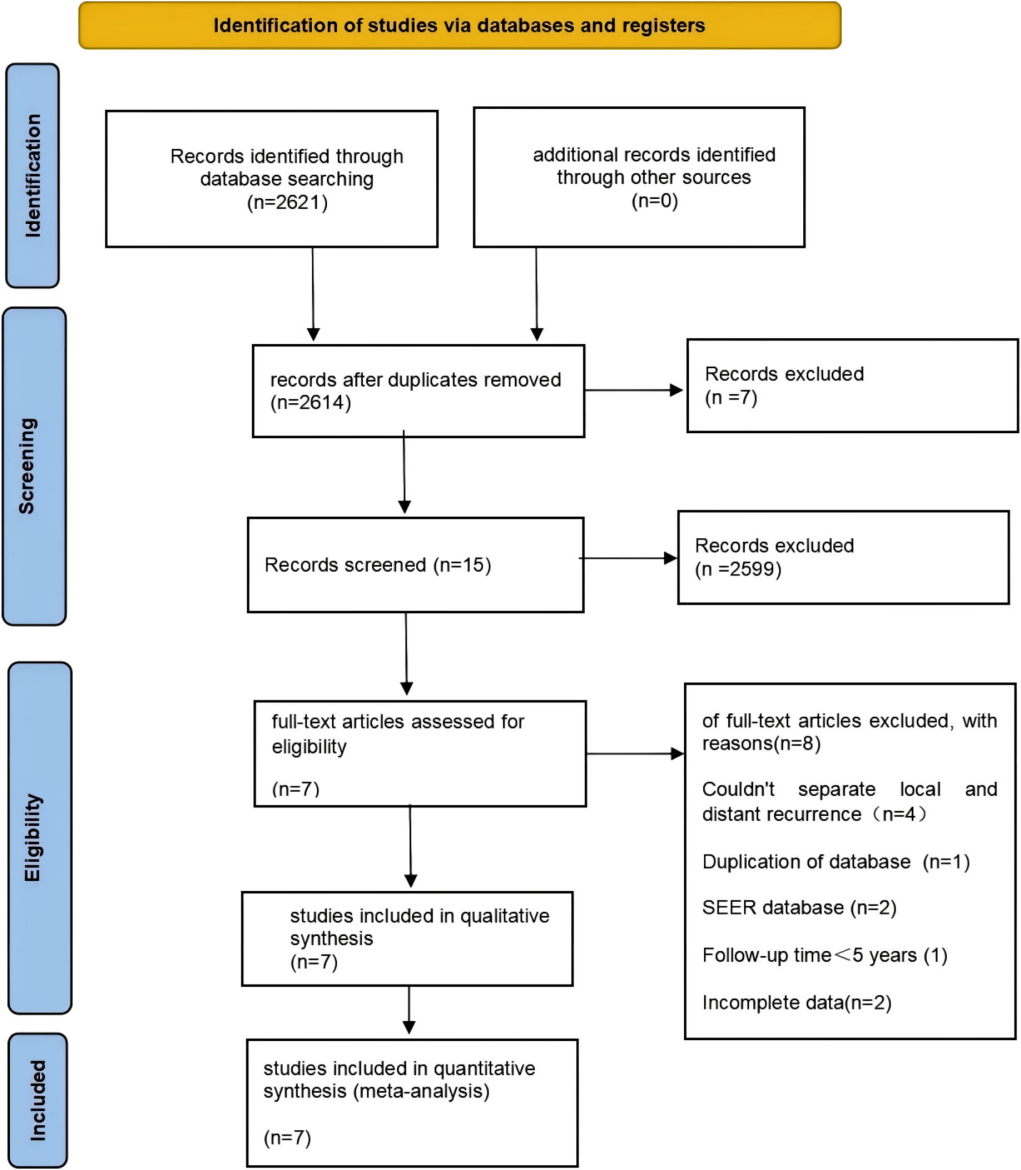
Preferred Reporting Items for Systematic Reviews and Meta-Analyses (PRISMA) flow diagram of the study selection process.

We systematically illustrated the literature selection process using a PRISMA flow diagram (**Figure 1**). Our initial screening identified 2, 621 studies published between 2015 and 2025. After deduplication and title/abstract screening, 127 studies proceeded to full-text assessment. We employed a dual independent screening approach. This process resulted in 7 retrospective cohort studies (16–22) meeting our predefined criteria. We excluded one duplicate cohort study derived from the same database. **Table 2** summarizes the characteristics of the included studies. All 7 studies featured retrospective cohort designs (16–22). Five studies enrolled Asian populations, while two involved European or North American populations. In total, the studies included 7, 731 patients. Among these, 2, 577 patients (33.3%) received PMRT, and 5, 205 patients (67.3%) did not receive PMRT. By definition, all studies included patients who had undergone systemic therapy. The table presents the primary chemotherapy regimens and the proportion of patients receiving them. The absolute LRR rate was 3.8% (56 events) in the PMRT group. This compared to 10.7% (210 events) in the no-PMRT group.

**Table 2:** Clinical trials studying patients with T1-T2 and 1-3 positive.

Author	Year	Region	Study type	Recruitment period	Study arms	Sample	median follow-up	Study arms	LRR(%)	HR	LRR_lower	LRR_upper	p-value	OS(%)	HR	OS_lower	OS_upper	p-value
Guo,X.Y	2021	China	retrospective	1997-2014	PMRT=1260	2520	63.8 (0.2-228.8)	PMRT=1260	4	0.5	0.38	0.64	0.001	93.8	0.68	0.55	0.84	0.001
					NO PMRT=1260			NO PMRT=1260	7.7					92.6				
Wang,S.L	2020	China	retrospective	2000-2014	PMRT=465	1986	68.5 (1-128)	PMRT=465	3.6	0.3	0.17	0.53	0.001	94.8	0.64	0.40	1.03	0.066
					NO PMRT=1521			NO PMRT=1521	6.6					94.9				
Kim,Y,J	2017	KR	retrospective	2006-2010	PMRT=130	663	69(1-114)	PMRT=130	3.1	0.83	0.29	2.39	0.734	98	1.14	0.43	3	0.793
					NO PMRT=584			NO PMRT=584	4.3					96				
Zeidan,Y.H	2018	USA	retrospective	1998-2000	PMRT=337	684	108(1-144)	PMRT=337	2.5	0.29	0.12	0.73	0.005	81.7	0.79	0.54	1.17	0.24
					NO PMRT=347			NO PMRT=347	6.5					78.3				
Tam,M.M	2017	USA	retrospective	2000-2003	PMRT=206	523	120(70.8-129.6)	PMRT=206	2	0.15	0.04	0.5	0.002	86	0.91	0.55	1.51	0.7
					NO PMRT=317			NO PMRT=317	9					84				
Miyashita,M	2017	Japan	retrospective	1999-2012	PMRT=100	658	87.6(2.4-213.6)	PMRT=100	/	0.266	0.746	0.064	0.0085	/	/	/	/	/
					NOPMRT=558			NOPMRT=558	/					/				
He,Z.Y	2015	China	retrospective	1998-2007	PMRT=79	697	65(6-144)	PMRT=79	11.1	0.073	0.01	0.531	0.01	93.1	/	/	/	0.646
					NO PMRT=618			NO PMRT=618	1.3					87.3				

### 4.2 Meta-analysis of PMRT Use and LRR

**Figure 2:**
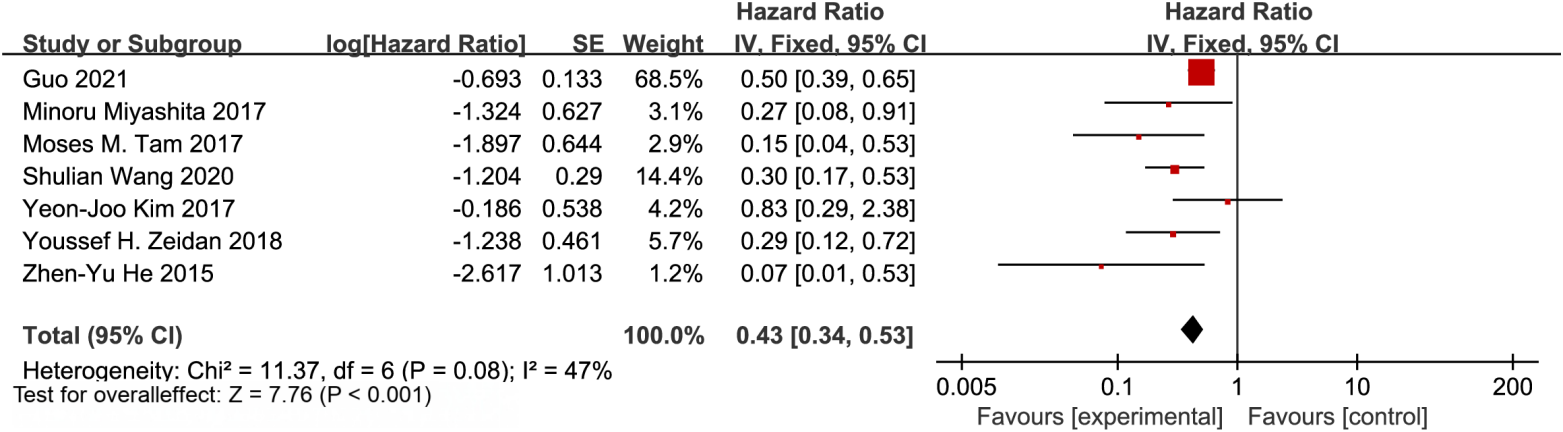
Forest plot of hazard ratios for locoregional recurrence comparing patients with T1–2N1M0 breast cancer receiving postmastectomy radiotherapy versus those not receiving radiotherapy.

We aimed to evaluate the therapeutic value of postmastectomy radiotherapy (PMRT) in breast cancer patients with T1/T2 stage tumors (size ≤5 cm) and 1–3 lymph node metastases. We pooled data from 7 retrospective studies (16–22). The studies showed significant heterogeneity (I² = 47%, p = 0.08). Therefore, we applied a random-effects model. Our analysis revealed that PMRT significantly reduced LRR risk in T1/T2N1M0 breast cancer patients. The pooled hazard ratio (HR) was 0.35 (95% CI: 0.23–0.53; p < 0.001) (**Figure 2**).

### 4.3 Meta-analysis of PMRT Use and OS

**Figure 3:**
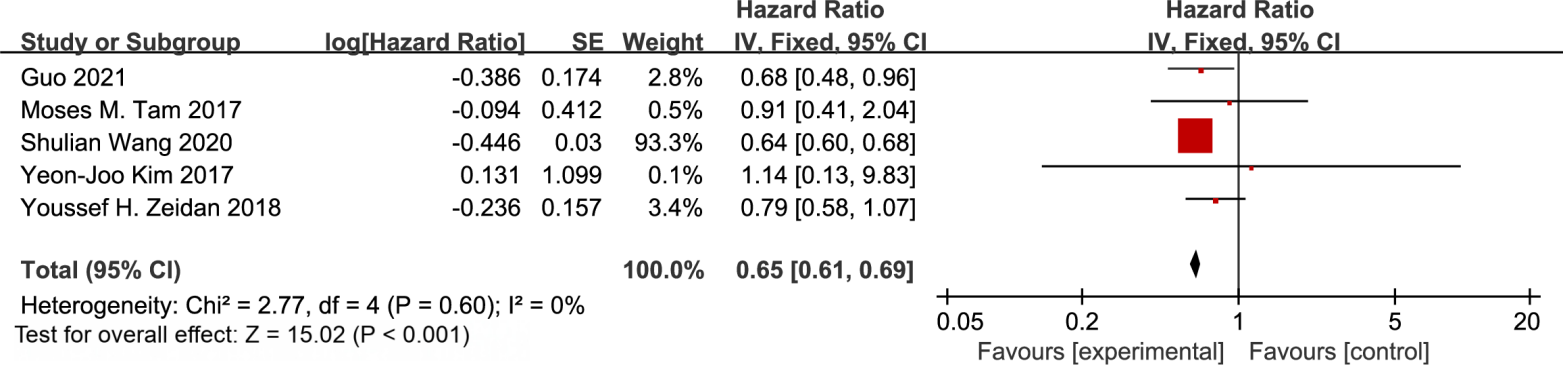
Forest plot of hazard ratios for overall survival comparing patients with T1–2N1M0 breast cancer receiving postmastectomy radiotherapy versus those not receiving radiotherapy.

We performed a pooled analysis of the 7 retrospective cohort studies (16–22). This analysis demonstrated a significant OS benefit in the PMRT group compared to the non-radiotherapy group. The hazard ratio (HR) was 0.65 (95% CI: 0.61–0.69; p < 0.001). We observed no heterogeneity among the studies (I² = 0%, p = 0.60) (**Figure 3**).

### 4.4 Meta-analysis of PMRT use and LRR in 5-year vs. 10-year patient subgroups

**Figure 4:**
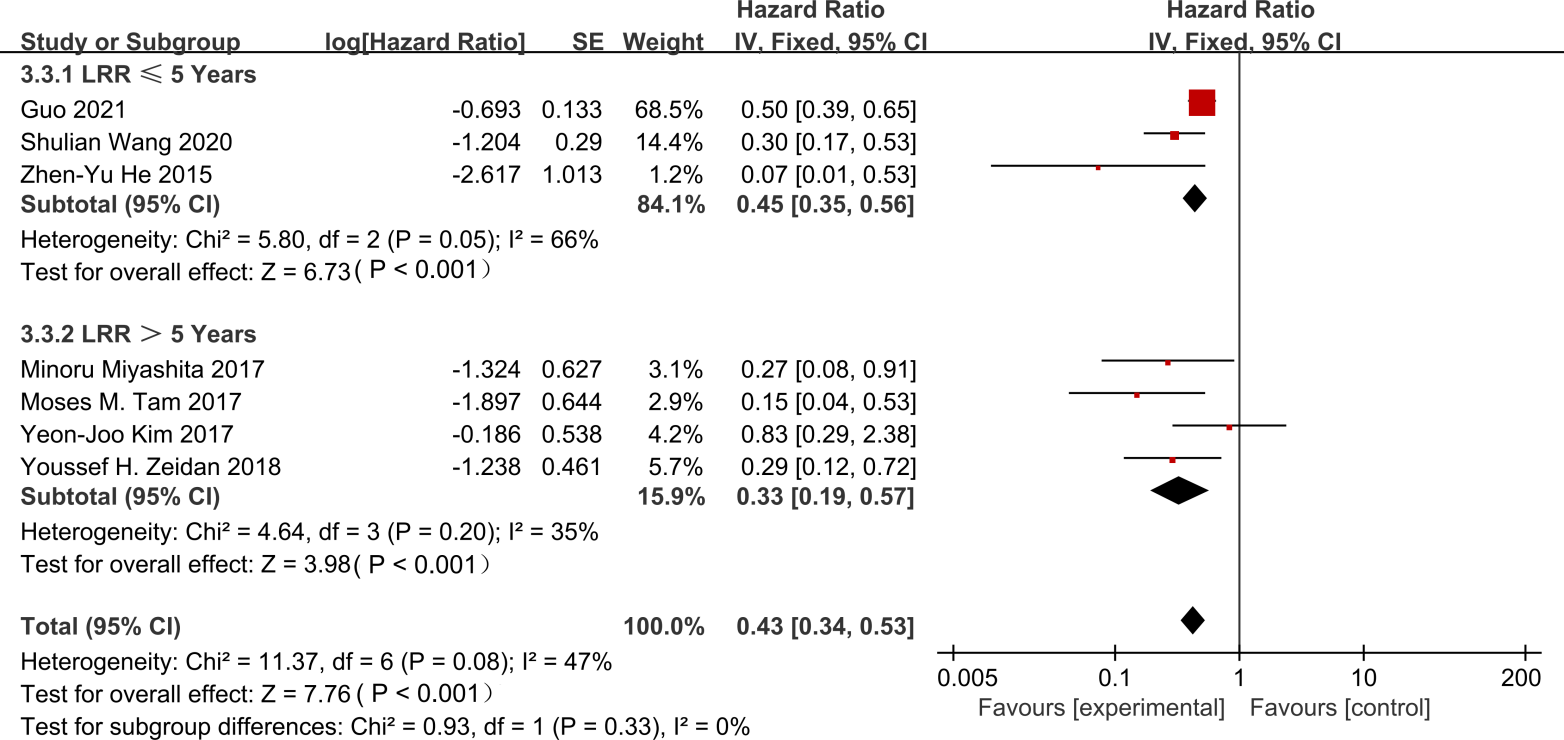
Subgroup analysis of hazard ratios for locoregional recurrence based on follow-up time (≤5 years vs. >5 years).

We performed a subgroup analysis to assess the impact of follow-up duration on the association between PMRT and LRR. Data from 7 studies (16–22) supported this analysis. The reduction in LRR risk was similar at 5 years and 10 years. The 5-year hazard ratio (HR) was 0.45 (95% CI: 0.35–0.56). The 10-year HR was 0.33 (95% CI: 0.19–0.57). There was no significant difference between these two time points (interaction p = 0.33). These findings suggest that the local control effect of PMRT remains consistent over time. All hazard ratios demonstrated statistical significance (p < 0.05) (**Figure 4**).

### 4.5 Meta-analysis of PMRT use and OS in 5-year vs. 10-year patient subgroups

**Figure 5:**
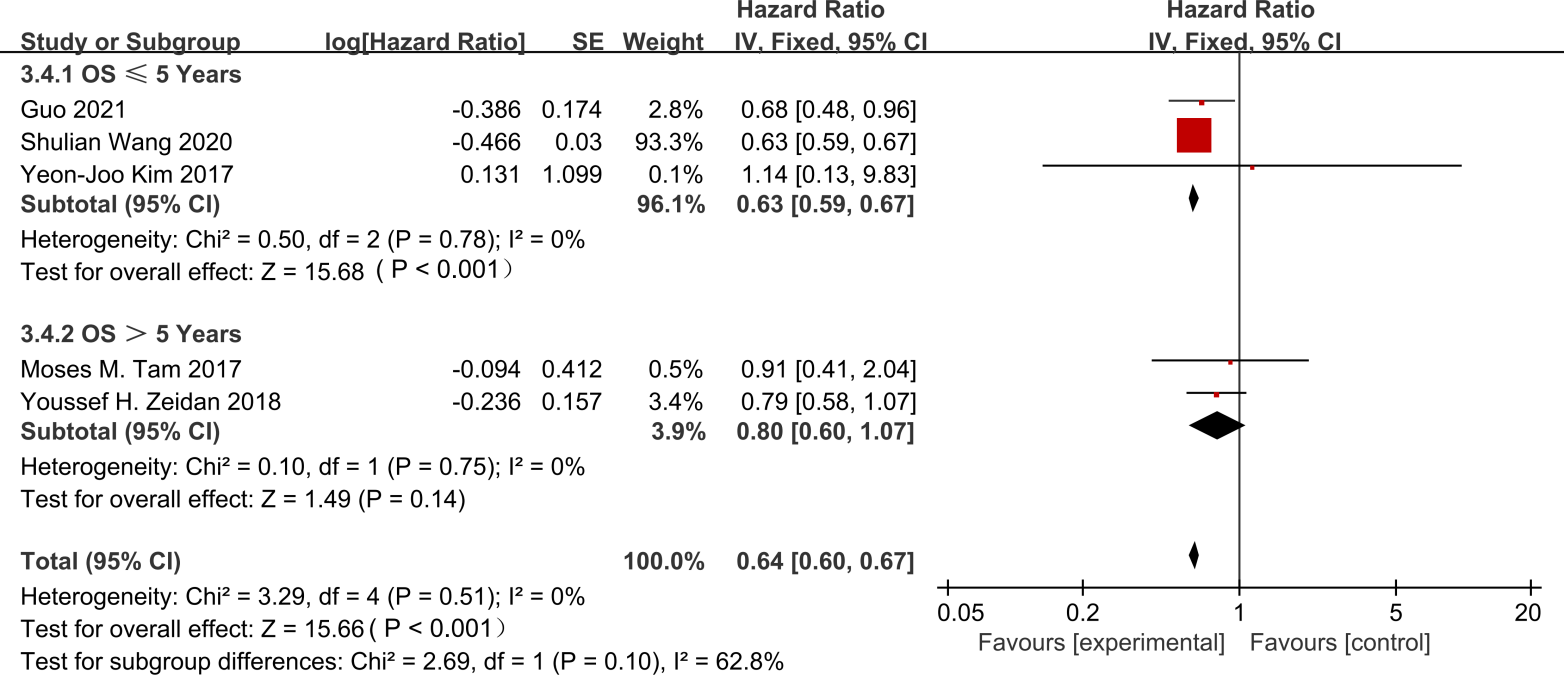
Subgroup analysis of hazard ratios for overall survival based on follow-up time (≤5 years vs. >5 years).

We conducted a subgroup analysis to assess the impact of follow-up time on the association between PMRT and OS. We observed significant heterogeneity among subgroups (I² = 62.8%, p = 0.10). This warranted the use of a random-effects model. Data from 5 studies (16, 17, 20–22) provided hazard ratios for this analysis. We stratified these by follow-up duration (≤5 years vs. >5 years). The between-subgroup heterogeneity was not statistically significant (I² = 62.8%, p = 0.10). PMRT demonstrated significant improvement in 5-year OS. The hazard ratio (HR) was 0.63 (95% CI: 0.59–0.67; p < 0.001). In contrast, the 10-year survival benefit did not reach statistical significance. The HR was 0.80 (95% CI: 0.60–1.07; p = 0.14) (**Figure 5**).

### 4.6 Modern systemic therapy

For breast cancer, current standard chemotherapy includes taxanes and anthracyclines. Taxanes include drugs like paclitaxel. Anthracyclines include drugs like doxorubicin. These are key parts of adjuvant systemic chemotherapy. For hormone receptor-positive breast cancer patients, endocrine therapy can lower local recurrence risk and improve long-term survival outcomes. Endocrine therapy includes drugs like tamoxifen and aromatase inhibitors (8, 9).

**Table 2** summarizes the use patterns of systemic adjuvant therapy in the included studies. All studies reported that their cohort patients received adjuvant systemic therapy.

Two studies are exceptions. They are Zeidan 2018 (BIG 02–98 trial, recruitment 1998–2000) and Miyashita 2017 (recruitment 1999–2012) (19, 22). In the other 5 studies (16–21), over 59.6% of patients received taxane-based or anthracycline/taxane combination chemotherapy. Notably, in Kim 2017 and Tam 2017 (17, 20), all patients got the standard regimen. This regimen is anthracycline-cyclophosphamide followed by taxane.

Five studies (16–19, 21) clearly recorded that HER2-positive patients used anti-HER2 agents. These agents include trastuzumab. The usage rate was between 0.7% and 18.2%. Endocrine therapy was the most widely used treatment. In the 6 studies that reported relevant data (16–19, 21, 22), the proportion of HR^+^ patients receiving standard endocrine therapy ranged from 71.3% to 100%.

These data show that the study populations in this meta-analysis generally received systemic adjuvant therapy. This therapy aligns with current clinical practice guidelines. But there was significant heterogeneity in treatment regimen composition and intensity across studies. This could be an important potential source of between-study heterogeneity.

## 5 Discussion

This meta-analysis included seven retrospective studies. The results show that for T1-2N1M0 breast cancer patients, PMRT significantly reduced the risk of LRR. We observed this outcome in both 5-year and 10-year follow-up subgroup analyses. This suggests the local control effect is stable over time (16–22). PMRT was also associated with improved 5-year OS. The hazard ratio was 0.63 (p < 0.001). But the 10-year OS benefit was not statistically significant. The hazard ratio was 0.80 (p = 0.14). There could be two reasons for this. First, distant metastasis risk increasingly dominates long-term survival outcomes (16, 17, 20–22). Second, the OS benefit itself may be time-dependent.

We observed moderate heterogeneity in the LRR analysis. The I² value was 47% (p = 0.08). This heterogeneity may come from differences in molecular subtype distribution across studies. Differences in radiotherapy techniques could also be a factor. We did not find heterogeneity in the OS analysis (I² = 0%) (16–22). Radiotherapy can prevent locoregional recurrence. Chemotherapy alone may not fully control such recurrence. This preventive effect helps bring survival benefits (23–25). Our results show that PMRT significantly reduces LRR risk. This aligns with the findings of EBCTCG, Overgaard, and others (2, 26–30). Also, meta-analyses by Chang, Li, and others (15, 31) show that in the modern systemic therapy era, PMRT still has clinical value in controlling local recurrence. But we also note an important point. The adjuvant chemotherapy regimens in early studies (2, 26) were inadequate by modern standards. Also, studies by Headon, Li, and others (28–32) reported that PMRT did not improve OS. This does not match our observed OS results. We observed a 5-year OS benefit in the PMRT group (HR = 0.63, p < 0.001). But the 10-year benefit was not significant (HR = 0.80, p = 0.14). This suggests that OS in the PMRT group may be time-dependent. This difference could come from several factors.

First, follow-up time may be insufficient. The EBCTCG study (2) noted that OS benefit for N1 patients may take over 10 years to appear. Their 20-year breast cancer mortality decreased by 7.9% (p = 0.01). In our study, three trials (16, 18, 19) had a median follow-up of only 5 years. This may underestimate long-term survival differences.

Second, modern systemic therapy has a major impact. Advances in systemic adjuvant therapy have significantly reduced distant metastasis risk (15, 33). PMRT may work together with systemic therapy. It clears subclinical disease in the chest wall and regional lymph nodes. These nodes include the supraclavicular and internal mammary lymph nodes (15, 34–37).

Third, population heterogeneity also plays a role.OS benefit may be diluted in low-risk subgroups. For example, patients with only 1 positive lymph node or T1 tumors. In contrast, high-risk subgroups show significant benefit. These subgroups include patients with 3 positive lymph nodes or T2 tumors (HR = 0.64, p = 0.02) (18, 38). It is worth noting that most studies did not show OS improvement. But reports by Huo and Overgaard (15, 26, 39) show statistically significant OS benefit. This suggests specific subgroups may benefit.These contradictions highlight two needs. First, risk stratification of the T1-T2N1M0 population is important (31, 40–47). Second, molecular subtyping is important. They emphasize the need for prospective studies with long-term follow-up. One important exception is the ongoing SUPREMO trial (47). It is expected to provide higher-level evidence to resolve these controversies.

### 5.1 Limitations of this study

All included studies in this research are retrospective cohort studies. No prospective studies or randomized controlled trials (RCTs) have been published yet. Retrospective studies have inherent design limitations. They are prone to selection bias. Residual confounding or unmeasured confounding is one problem. Heterogeneity in interventions across studies is another problem. This includes differences in systemic therapy, radiotherapy regimens, and follow-up time. Overall, these factors reduce the reliability of the pooled effect size.

According to our GRADE assessment (**Table 3**), the overall evidence quality for main outcomes is ‘moderate’. These outcomes are LRR and OS. Based on this evidence, the robustness of our conclusion is limited.

**Table 3:** Modern systemic therapy.

Author	Year	Region	Study type	Recruitment period	Adjuvant systemic therapy	NOS score
Guo, X.Y	2021	China	retrospective	1997-2014	A+T(59.6%)	7
					hormonal therapy(83.2%)	
					anti-HER2 targeted therapy(18.2%)	
Wang, S.L (21)	2020	China	retrospective	2000-2014	A+T(69.3%)	7
					hormonal therapy(78.2%)	
					anti-HER2 targeted therapy(9.2%)	
Kim, Y.J (17)	2017	KR	retrospective	2006-2010	AC-T(95.8%)	8
					hormonal therapy(78.2%)	
					anti-HER2 targeted therapy(14.6%)	
Zeidan, Y.H (22)	2018	USA	retrospective	1998-2000	A+T(67.0%)	6
					hormonal therapy(71.3%)	
					anti-HER2 targeted therapy(0%)	
Tam, M. M (20)	2017	USA	retrospective	2000-2003	AC-T(100%)	9
					hormonal therapy(Not Specified)	
					anti-HER2 targeted therapy(Not Specified)	
Miyashita, M (19)	2017	Japan	retrospective	1999-2012	A(31%)	7
					hormonal therapy(78%)	
					anti-HER2 targeted therapy(4%)	
He, Z.Y (18)	2015	China	retrospective	1998-2007	A/T(92.7%)	6
					hormonal therapy (100%)	
					anti-HER2 targeted therapy(0.7%)	

During data extraction, we found that most studies did not report outcomes by molecular subtype. This made it difficult to manually perform subtype-level stratified analysis. Also, some studies only provided overall hazard ratios. They did not provide results for specific subtypes. In this case, pooled subgroup analysis by molecular subtype would likely introduce significant bias.

We could not perform stratified analysis to address population heterogeneity. We also could not explore factors that might affect LRR and OS. In retrospective datasets, the decision to give PMRT may be influenced by clinician preference. This could introduce residual confounding and bias the results.

Also, some key prospective trials have not reported their outcomes yet. One example is the ‘Selective Use of Postoperative Radiotherapy After Mastectomy’ (SUPREMO) trial. This limits our ability to fully assess the long-term safety and efficacy of PMRT.

## 6 Conclusion

In conclusion, this meta-analysis of T1-T2N1M0 breast cancer patients demonstrates that PMRT significantly reduces the risk of LRR and improves OS, providing robust evidence for its clinical application in this population. However, future clinical decisions should incorporate molecular subtyping, dynamic risk models, and modern systemic therapies to optimize PMRT use, define indications for specific clinical scenarios, and assess the long-term safety of PMRT omission through prospective studies.

## Data Availability

The original contributions presented in the study are included in the article/**Supplementary Material**. Further inquiries can be directed to the corresponding author.
