# Supplementary figures and images for: Can radiotherapy be omitted in T1-2N1 breast cancer patients after mastectomy without neoadjuvant therapy?
Source: Front Oncol. 2026 Jan 23;15:1726994. doi: 10.3389/fonc.2025.1726994 (PMC12875952; doi:10.3389/fonc.2025.1726994)

# Funnel Plot for LRR

Egger P-value: 0.254

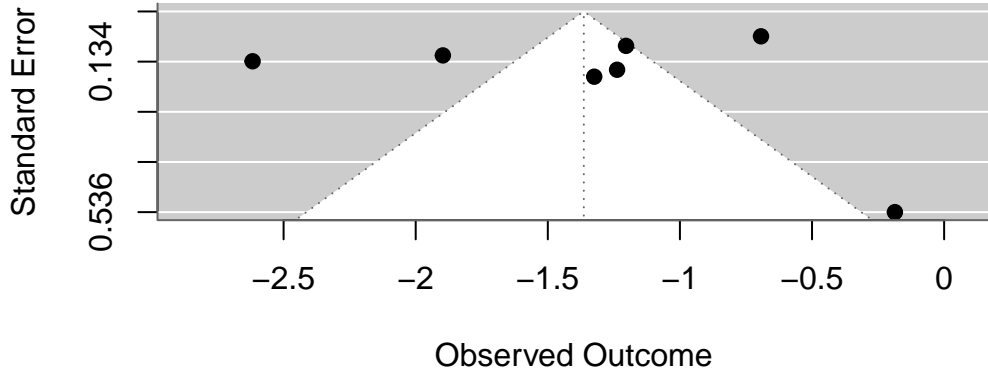

Supplement: Supplementary file 1 [file DataSheet1.pdf]

# Funnel Plot for OS

Egger P-value: 0.223

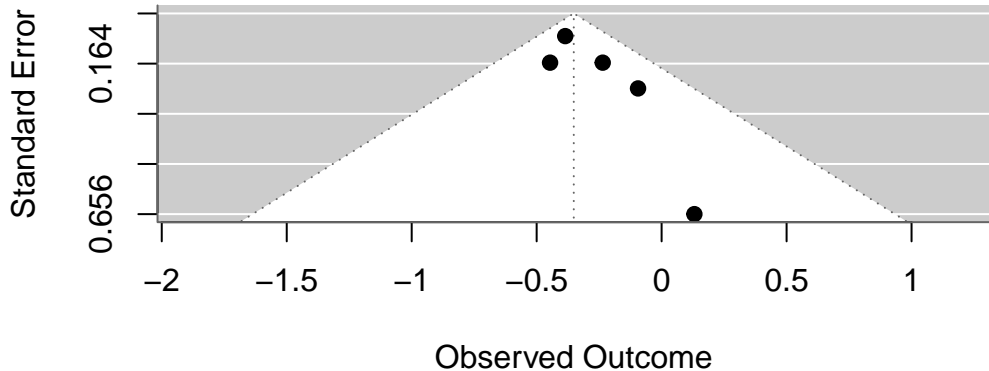

Supplement: Supplementary file 2 [file DataSheet2.pdf]
